# Bioactive Composition, Antioxidant Activity, and Anticancer Potential of Freeze-Dried Extracts from Defatted Gac (*Momordica cochinchinensis* Spreng) Seeds

**DOI:** 10.3390/medicines5030104

**Published:** 2018-09-18

**Authors:** Anh V. Le, Tien T. Huynh, Sophie E. Parks, Minh H. Nguyen, Paul D. Roach

**Affiliations:** 1 School of Environmental and Life Sciences, University of Newcastle, Ourimbah, NSW 2258, Australia; sophie.parks@dpi.nsw.gov.au (S.E.P.); Minh.Nguyen@newcastle.edu.au (M.H.N.); Paul.Roach@newcastle.edu.au (P.D.R.); 2 Faculty of Bio-Food Technology and Environment, University of Technology (HUTECH), Ho Chi Minh City 700000, Vietnam; 3 School of Science, RMIT University, Bundoora, VIC 3083, Australia; tien.huynh@rmit.edu.au; 4 Central Coast Primary Industries Centre, NSW Department of Primary Industries, Ourimbah, NSW 2258, Australia; 5 School of Science and Health, Western Sydney University, Penrith, NSW 2751, Australia

**Keywords:** *Momordica cochinchinensis*, Gac, seeds, saponins, trypsin inhibitors, phenolics, anticancer, antioxidant, extraction, freeze dried extract

## Abstract

**Background:** Gac (*Momordica cochinchinensis* Spreng) seeds have long been used in traditional medicine as a remedy for numerous conditions due to a range of bioactive compounds. This study investigated the solvent extraction of compounds that could be responsible for antioxidant activity and anticancer potential. **Methods:** Defatted Gac seed kernel powder was extracted with different solvents: 100% water, 50% methanol:water, 70% ethanol:water, water saturated butanol, 100% methanol, and 100% ethanol. Trypsin inhibitors, saponins, phenolics, and antioxidant activity using the 2,2’-azino-bis(3-ethylbenzothiazoline-6-sulfonic acid) diammonium salt (ABTS), the 2,2-diphenyl-1-picrylhydrazyl (DPPH) and the ferric reducing antioxidant power (FRAP) assays; and anticancer potential against two melanoma cancer cell lines (MM418C1 and D24) were analysed to determine the best extraction solvents. **Results:** Water was best for extracting trypsin inhibitors (581.4 ± 18.5 mg trypsin/mg) and reducing the viability of MM418C1 and D24 melanoma cells (75.5 ± 1.3 and 66.9 ± 2.2%, respectively); the anticancer potential against the MM418C1 cells was highly correlated with trypsin inhibitors (r = 0.92, *p* < 0.05), but there was no correlation between anticancer potential and antioxidant activity. The water saturated butanol had the highest saponins (71.8 ± 4.31 mg aescin equivalents/g), phenolic compounds (20.4 ± 0.86 mg gallic acid equivalents/g), and antioxidant activity, but these measures were not related to anticancer potential. **Conclusions:** Water yielded a Gac seed extract, rich in trypsin inhibitors, which had high anticancer potential against two melanoma cell lines.

## 1. Introduction

*Momordica cochinchinensis* Spreng, commonly called Gac, is a plant species of the family *Cucurbitaceae*, which is also known as red melon, baby jackfruit, spiny bitter gourd, sweet gourd, and cochinchin gourd. It is native to Southeast Asia and is commonly grown as a food crop in Vietnam, Thailand, Laos, Myanmar, and Cambodia [1,2]. From the food and commercial perspectives, the most commonly used part of the mature fruit is the red flesh surrounding the seeds, called the aril, which is traditionally used as a colourant in rice or, more recently, as a material that is processed into functional food ingredients or supplements [3].

The seeds are not eaten; they are removed from the aril and are mostly considered as waste [3,4]. However, in traditional medicine, Gac seeds are purported to have an array of therapeutic effects on a variety of conditions, such as fluxes, liver and spleen disorders, haemorrhoids, wounds, bruises, swelling, and pus [2,5]. In modern research, several constituents have been identified, which could be involved in the putative medicinal properties of Gac seeds, including trypsin inhibitors (e.g., MCoTI-I, MCoTI-II and MCoTI-III) [6,7,8], saponins (e.g., Momordica Saponin I and Momordica Saponin II) [9,10], and phenolic compounds (e.g., gallic acid and *p*-hydroxybenzoic acid) [11]. Gac seed extracts have been linked with many medicinal properties, such as gastroprotective [12,13,14], anti-inflammatory [15,16], anticancer [17], and antitumor [18] activities. Most of these properties are linked to the seed’s saponins [12,13,15,16]. Furthermore, karounidiol, a triterpenoid compound, likely a saponin, present in Gac seeds [19], possess cytotoxic activity against human cancer cell lines [20]. Protease inhibitors, like the trypsin inhibitors in Gac seeds [7,8,21], have diverse biochemical functions [4,22], including acting as anticancer agents by inhibiting the growth of transformed cells [23,24,25,26]. Some trypsin inhibitors, such as those from *Cajanus cajan* and *Phaseolus limensis*, possess antioxidant, anti-inflammatory, and anti-bacterial activity [27].

There is limited information on how to best recover the bioactive compounds from Gac seeds, particularly to optimise anticancer potential. Efficient extraction and preservation methods are important for their immediate and long-term use. For any given plant bioactive, extractable yield depends on the extraction solvent, the chemical nature of the targeted component, and the characteristics of the extraction procedure. When other factors are kept constant, the extraction solvent plays a key role in obtaining the desired quality and quantity of the target constituents [28,29]. The choice of solvent is mainly based on the chemical properties of the targeted compounds, such as their polarity or hydrophobicity.

Due to the hydrophilic or amphiphilic nature of the trypsin inhibitors, phenolics, and saponins in Gac seeds, aqueous solvents and the low alcohols are likely to be the best extraction solvents for these bioactive compounds. They are also the safest and the most environmentally-friendly solvents for the extraction of bioactive compounds from plant materials [30,31]. For drying after solvent extractions, numerous methods have been developed; however, freeze drying is considered to be superior for preserving the medicinal qualities of botanical extracts and it is therefore widely used [32]. Additionally, to make it easier for the extraction of trypsin inhibitors [33] and to avoid interference of sticky oil during the freeze-drying procedure, the Gac seeds need to be defatted before they are extracted with solvents.

Thus, this study aimed to investigate the effectiveness of different solvents (water, alcohols, and mixtures) to extract the bioactive compounds of interest (trypsin inhibitors, saponins, and phenolics) from defatted Gac seed kernel powder. The relationships between the extracted compounds and the antioxidant activity and the anticancer potential of the extracts were also investigated.

## 2. Materials and Methods

### 2.1. Materials

#### 2.1.1. Solvents, Reagents, and Chemicals

Solvents (ethanol, methanol and butanol) and chemicals (vanillin, sulphuric acid and potassium persulfate) were purchased from Merck (Bayswater, VIC, Australia). Folin-ciocalteu’s phenol reagent, anhydrous sodium carbonate, sodium nitrile, ferric chloride, gallic acid, catechin, 2,4,6-tris(2-pyridyl)-s-triazine, (±)-6-hydroxy-2,5,7,8-tetramethylchromane-2-carboxylic acid (trolox), aescin, 2,2-diphenyl-1-picrylhydrazyl (DPPH), 2,2′-azino-bis(3-ethylbenzothiazoline-6-sulfonic acid) diammonium salt (ABTS), trypsin (type I) from bovine pancreas, benzyl-DL-arginine-para-nitroanilide (BAPNA), Tris, and dimethylsulfoxide (DMSO) were purchased from Sigma-Aldrich Co. (Castle Hill, NSW, Australia). Sodium acetate trihydrate was purchased from Government Stores Department (Sydney, NSW, Australia). Aluminium chloride was a product of J. T. Baker Chem. Co. (Thermo Fisher Scientific, North Ryde, NSW, Australia). Acetic acid was a product of BDH Laboratory Supplies (Bio-Strategy, Tingalpa, QLD, Australia). Sodium hydroxide was a product of Ajax FineChem (Thermo Fisher Scientific, North Ryde, NSW, Australia) and hydrochloride acid a product of Lab-Scan Ltd. (Bacto, Mt Pritchard, NSW, Australia).

Human melanoma MM418C1 (mutated BRAF oncogene), referred to as C1 melanoma cells, and D24 (wild type BRAF oncogene) and human keratinocyte (HaCat) cell lines were provided by the School of Health and Biomedical Sciences, RMIT University (Bundoora, VIC, Australia). RPMI-1640 media, streptomycin, and penicillin were Gibco products (Thermo Fisher Scientific, North Ryde, NSW, Australia). Fetal bovine serum (FBS) was from Serana (Melbourne, VIC, Australia).

#### 2.1.2. Gac Seeds

Gac seeds, from accession VS7 as classified by Wimalasiri et al. [1], were collected from 450 kg of fresh Gac fruit. These fruits were bought at Gac fruit fields in Dong Nai province, Ho Chi Minh City, Vietnam (Latitude: 10.757410; Longitude: 106.673439). After their separation from the fresh fruit, the seeds were vacuum dried at 40 °C for 24 h to reduce moisture and increase the crispness of the shell, which facilitated shell removal. The dried seeds were de-coated to get the kernels, which were then packaged in vacuum-sealed aluminium bags and stored at −18 °C until used.

Preparation of Defatted Gac Seed Kernel Powder

Defatted Gac seed kernel powder was prepared as described by Le et al. [34]. Briefly, the Gac seed kernels were ground in an electric grinder (100 g ST-02A Mulry Disintegrator, Taiwan Machinary, Sydney, NSW, Australia) to a powder that could pass through a sieve of 1.4 mm. The powder was then freeze-dried using a Dynavac FD3 Freeze Dryer (Dynapumps, Seven Hills, NSW, Australia) for 48 h, at −45 °C under vacuum at a pressure loading of 10^−2^ mbar (1 Pa), to reduce the moisture content to 1.21 ± 0.02%. The powder was then defatted using three 30-min extractions with hexane at a powder to hexane ratio of 1:5 *w*/*v*. The resulting slurry was suction filtered and the residue (defatted meal) was air-dried for 12 h and stored in a desiccator at ambient room temperature until used. The moisture content of the defatted powder, measured using a Shimadzu MOC63u moisture analyser (Rydalmere, NSW, Australia), was 8.61 ± 0.15%.

### 2.2. Methods

#### 2.2.1. Extraction

Based on a previous study [34], six solvents (deionised water, 100% methanol, 50% methanol in water, 100% ethanol, 70% ethanol in water, and 90% *n*-butanol in water), were used for the extraction of bioactive compounds from the defatted and dried Gac seed kernel powder. Twenty grams of the powder were added to 400 mL of each solvent and the suspension was kept under constant magnetic stirring for 30 min at 40 ± 1 °C. Following extraction, the mixtures were filtered through two layers of cheese cloth and then through a Whatman No. 1 filter paper (Thermo Fisher Scientific, North Ryde, NSW, Australia), and the clear filtrates were collected in 500 mL evaporating flasks. Triplicate extractions were done for each solvent.

#### 2.2.2. Freeze Drying Extracts

**Figure 1:**
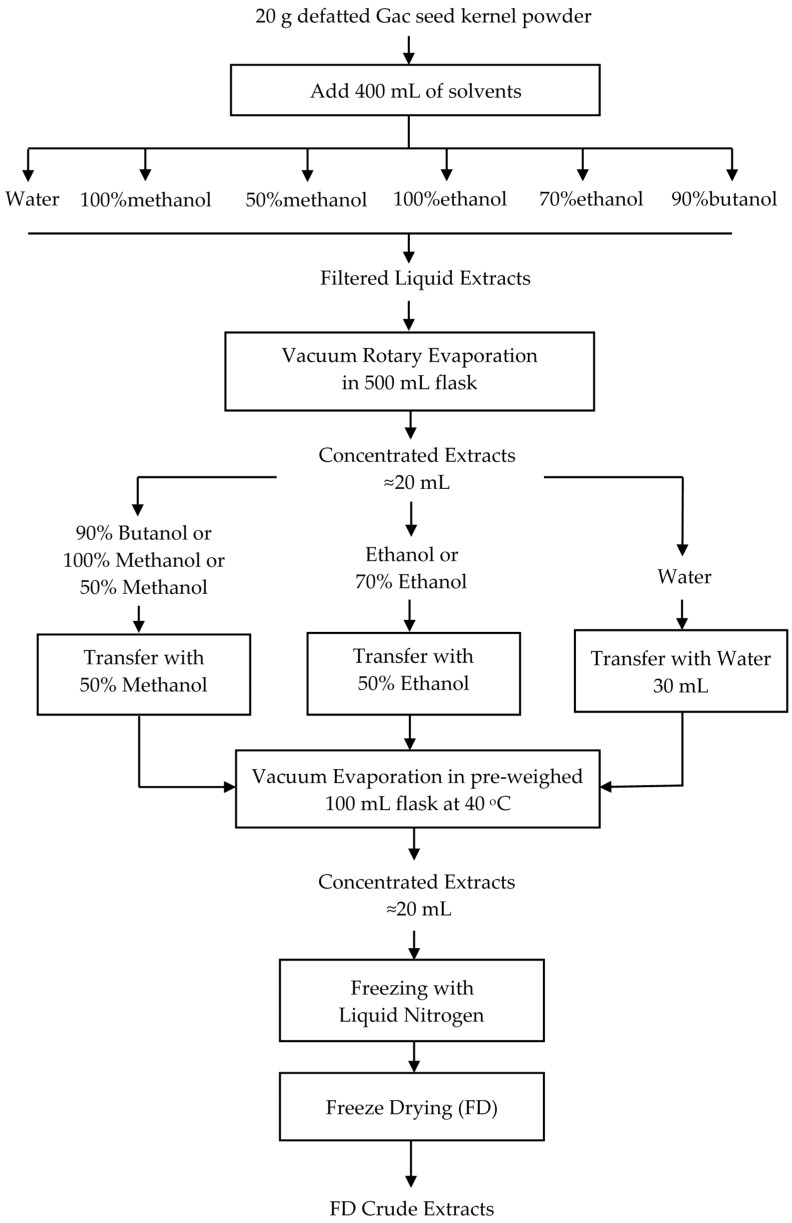
Procedure for producing freeze dried (FD) crude extracts from defatted Gac seed kernel powder.

The filtrates collected from the extractions were freeze-dried into powders as summarized in Figure 1. First, the liquid extracts were concentrated using a rotary evaporator (Buchi Rotavapor B480, Buchi Australia, Noble Park, VIC, Australia) at 40 °C under vacuum until thick but not completely dried in the 500 mL evaporating flasks. Then, to transfer the extracts into pre-weighed 100 mL evaporating flasks, three different solvents were used. For each of the butanol, methanol, and 50% methanol extracts, 50 mL of 50% methanol in water was used, for each of the ethanol and 70% ethanol extracts, 50 mL of 50% ethanol in water was used, and for the water extract, 30 mL of water was used. The suspensions were mixed well, and the evaporation resumed until around 20 mL of the concentrated extracts were left. The concentrates were then frozen using liquid nitrogen before freeze drying with a BenchTop Pro freeze dryer (Scitek, Lane Cove, NSW, Australia) at −60 °C and 30 mbar for 48 h. The flasks with residue were placed in a desiccator and quickly weighed. The difference in weight between the flasks with residue and the empty flasks was taken to be the mass recovered for each extract. These freeze dried (FD) crude extracts were then stored in air-tight containers at −20 °C for use within 3 months.

#### 2.2.3. Determination of Extractable Yield

To determine the extractable yield, 10 mL of each filtered liquid extract, resulting from Section 2.2.1, was transferred into a tared flat-bottomed glass vial and then dried at 105 °C with a vacuum pressure of 60 kPa for 24 h in a vacuum oven (Thermoline, Wetherill Park, NSW, Australia) until a constant weight was achieved. These vials were cooled in a desiccator for 30 min and weighed. The extractable yield was calculated, in g dried extract per 100 g of dried defatted Gac seed kernel powder, using Equation (1), where EY was the extractable yield, DE (g) was the mass of dried extract after the vacuum oven drying, 40 was the ratio of the 10 mL used for the vacuum oven drying to the 400 mL originally used for the extract, and DS (g) was the mass of dried defatted Gac seed kernel powder used for the extraction.

(1) $$\;\mathrm{EY}\;\left(g/100\;g\right)=\;\frac{\mathrm{DE}\times 40}{\mathrm{DS}}\times 100\;$$

#### 2.2.4. Determination of Dry Mass Yield

Dry mass yield was defined as the amount (g) of FD crude extract, produced as described in Section 2.2.2, per 100 g of dried defatted Gac seed kernel powder. Equation (2) was used to calculate the dry mass yield (DM), in which FD (g) was the weight of the FD crude extract, DS (g) was the mass of dried defatted Gac seed kernel powder used for the extraction, and V was the volume of the filtrate collected after extraction.

(2) $$\;\mathrm{DM}\;\left(g/100\;g\right)=\;\frac{\mathrm{FD}\;\times V/\left(V-10\right)}{\mathrm{DS}}\times 100\;$$

#### 2.2.5. Determination of Trypsin Inhibitor Activity (TIA)

The TIA assay was performed as described by Makkar et al. [35] except that the absorbance was measured at 385 nm, as suggested by Stauffer [36], instead of at 410 nm.

##### Reagent Preparation

*Substrate solution*: A substrate solution of 92 mM BAPNA was prepared as follows. First, 40.0 mg BAPNA was dissolved in 1.00 mL DMSO and diluted to 100 mL with 0.05 M Tris-buffer (pH 8.2) containing 0.02 M CaCl_2_ pre-warmed to 37 °C. This solution was prepared daily and kept at 37 °C while in use.

*Trypsin solution*: 20.0 mg of trypsin (type I) from bovine pancreas was dissolved in 1 mM HCl to make 1 L and stored at 4 °C for use within a week. In the analytical procedure with 92 mM BAPNA, this solution gave an absorbance value in the range of 0.900 ± 0.010 after subtracting the reagent blank at 385 nm.

##### Determination of TIA

Each FD crude extract from Section 2.2.2 (Figure 1) was dissolved in water at a concentration to give an inhibition of Trypsin between 40% and 60% and the assay was setup as shown in Table 1 with four test tubes prepared for each FD crude extract. All the prepared test tubes were kept in a water bath at 37 °C for 10 min to promote the formation of an enzyme–inhibitor complex and then 5.0 mL of BAPNA solution, pre-warmed to 37 °C, was added into each tube and the tubes were incubated in a water bath at 37 °C for 10 min. One mL of 30% acetic acid solution was added to each tube to stop the reaction. Then, 2.0 mL of trypsin solution was added into each reagent and sample blank (Table 1). After thorough mixing, the absorbance of the reaction mixture due to the release of *p*–nitroaniline was measured at 385 nm.

##### Calculation

The change in absorbance (A_I_) due to the trypsin inhibitor per mL of diluted extract was (A_b_ − A_a_) − (A_d_ − A_c_), where the subscripts referred to tubes (a) to (d) in Table 1. Since 1 µg of trypsin gave an absorbance of 0.0190, the weight of trypsin inhibited per mL of extract was A_I_/0.019 µg. From this value, The TIA of the FD crude extracts was calculated using Equation (3) and expressed as mg of pure trypsin inhibited per mg of FD crude extract.

(3) $$\;\mathrm{TIA}=\;\frac{A_{I}}{0.019\;\times S\;\times \left(1-\frac{m\%}{100}\right)}\;$$

where,

A_I_: Change in absorbance due to inhibition per 1 mL of extract;
A_I_ = (A_b_ – A_a_) – (A_d_ – A_c_), subscripts as per Table 1;
S: Weight (mg) of the FD crude extract dissolved in 1 mL;
m%: Moisture content of the FD crude extract powder.

#### 2.2.6. Determination of Total Saponin Content (TSC)

The FD crude extracts from Section 2.2.2 were dissolved in water at a concentration of 2 mg/mL and vortexed before the TSC was determined according to Tan et al. [37] with some modifications. Briefly, 0.25 mL of each extract was mixed with 0.25 mL of 8% (*w*/*v*) vanillin solution and 2.5 mL of 72% (*v*/*v*) sulphuric acid. The mixture was vortexed and incubated in a water bath at 60 °C for 15 min and then cooled on ice for 10 min. The absorption of the mixture was measured at 560 nm using a Cary 60 UV-Vis spectrophotometer (Agilent Technologies, Mulgrave, VIC, Australia). Aecsin was used as a standard and the results were expressed as milligram aecsin equivalents (AE) per gram of the FD crude extract powder (mg AE/g).

#### 2.2.7. Determination of Total Phenolic Content (TPC)

The FD crude extracts from Section 2.2.2 were dissolved in water at a concentration of 2 mg/mL and vortexed before the TPC was determined according to the method of Tan et al. [38] with some modifications. Briefly, 0.5 mL of each extract was mixed with 2.5 mL of 10% (*v*/*v*) Folin–Ciocalteu reagent in water and incubated at room temperature for 2 min to equilibrate. Then, 2 mL of 7.5% (*w*/*v*) sodium carbonate solution in water was added and the mixture was incubated at ambient temperature for 1 h. The absorption of the reaction mixture was measured at 765 nm using a Cary 60 UV-Vis spectrophotometer. Gallic acid was used as a standard and the results were expressed as milligram gallic acid equivalents (GAE) per gram dry weight of the FD crude extract powder (mg GAE/g).

#### 2.2.8. Determination of Antioxidant Capacity

The FD crude extracts from Section 2.2.2 were dissolved in water at the concentration of 2 mg/mL and mixed before analysing antioxidant capacity using three assays: the 2,2’-azino-bis(3-ethylbenzothiazoline-6-sulfonic acid) diammonium salt (ABTS), the 2,2-diphenyl-1-picrylhydrazyl (DPPH) and the ferric reducing antioxidant power (FRAP).

##### DPPH

The DPPH assay measures the total free radical scavenging capacity of the extracts. The assay was performed as described by Tan et al. [38]. A stock solution of 0.6 M DPPH in methanol was prepared and kept at −20 °C until use. The working solution was prepared by mixing 10 mL of stock solution with 45 mL of methanol to obtain an absorbance of 1.1 ± 0.02 units at 515 nm using a spectrophotometer. Each extract (0.15 mL) was mixed with 2.85 mL of the working solution and the mixture was allowed to stand for 3 h, after which the absorption was measured at 515 nm using a Cary 60 UV-Vis spectrophotometer. Trolox was used as a standard and the results were expressed as milligram Trolox equivalents (TE) per gram of the FD crude extract powder (mg TE/g).

##### ABTS

The ABTS assay measures the total free radical scavenging capacity of the extracts. The assay was performed as described by Tan et al. [38] with slight modifications. Stock solutions of 7.4 mM ABTS and 2.6 mM potassium persulfate were freshly prepared or kept at 4 °C in a dark bottle for use within a month, respectively. A fresh working solution was prepared for each assay by mixing equal quantities of the two stock solutions and incubated for 15 h to 16 h in the dark at ambient temperature. Then, 1 mL of the working solution was diluted with approximately 30 mL of methanol to obtain an absorbance of 1.1 ± 0.02 units at 734 nm using a Cary 60 UV-Vis spectrophotometer. Each extract (0.15 mL) was mixed with 2.85 mL of the working solution and the mixture was incubated for 2 h in the dark at ambient temperature. The absorption of the reaction mixture was measured at 734 nm using a Cary 60 UV-Vis spectrophotometer. Trolox was used as a standard and the results were expressed as milligram Trolox equivalents (TE) per gram of the FD crude extract powder (mg TE/g).

##### FRAP

The FRAP assay measures the ferric reducing power. The assay was performed following the method of Thaipong et al. [39], based on the increase in absorbance at 593 nm. A fresh FRAP working solution was initially prepared by mixing 300 mM acetate buffer (pH 3.6), 10 mM iron reagent (TPTZ) in 40 mM HCl, and 20 mM FeCl_3_•6H_2_O in the ratio of 10:1:1 (*v*/*v*/*v*). The fresh working solution was warmed to 37 °C before using. Each extract (0.15 mL) was mixed with 2.85 mL of the working FRAP solution and the mixture was incubated at ambient temperature in the dark for 30 min before its absorbance was measured at 593 nm using a Cary 60 UV-Vis spectrophotometer. Trolox was used as a standard and the antioxidant capacity of each sample, based on its ability to reduce ferric ions, was expressed as milligram Trolox equivalents (TE) per gram of the FD crude extract powder (mg TE/g).

#### 2.2.9. Determination of Cytotoxicity

##### Cell Lines and Culture

The human melanoma MM418C1 (C1, wild type BRAF oncogene) and D24 (mutated BRAF oncogene) cell lines were maintained in RPMI-1640 media supplemented with 10% (*v*/*v*) FBS, 1% (*v*/*v*) streptomycin and penicillin at 37 °C in 5% CO_2_. HaCat keratinocytes were used as normal untransformed cells and grown in the same media.

##### In Vitro Cytotoxicity Assay

All cells were seeded in 96 well plates (Greiner Bio-One, Labfriend, Sydney, NSW, Australia), 5000 cells/well along with 100 μL of fresh media. The FD crude extracts from Section 2.2.2 were dissolved in RPMI-1640 cell culture media at a concentration of 2 mg/mL and UV-sterilised for 10 min in a laminar flow hood before use on cells. The cells were allowed to attach for 4 h before being treated with 10 μL of the extract and incubated for 48 h.

The effect of the extracts on cell growth was determined using the CCK-8 (Cell Counting Kit-8) assay (Sigma-Aldrich, St Louis, MO, USA). The assay measured cytotoxicity based on the conversion of a water-soluble tetrazolium salt, 2-(2-methoxy-4-nitrophenyl)-3-(4-nitrophenyl)-5-(2,4-disulfophenyl)-2*H*-tetrazolium, monosodium salt (WST-8), to a water-soluble formazan dye upon reduction by dehydrogenases in the presence of an electron carrier [40].

To determine cell viability, 10 μL of CCK-8 solution was added to each well of the 96-well plate containing treated and control samples. The plates were incubated at 37 °C for 2 h and the absorbance was measured spectrophotometrically at 450 nm using a CLARIOstar^®^ High Performance Monochromator Multimode Microplate Reader (BMG LABTECH, Mornington, NSW, Australia) and the results were analysed using the MARS data analysis software (version 3.00R2, BMG LABTECH, Mornington, NSW, Australia). The data were presented as a proportional viability (%) by comparing the treated cells with the untreated cells (control) using Equation (4):

(4) $$\;\mathrm{Cell}\;\mathrm{viability}=\frac{At-Ab}{Ac-Ab}\times 100\;$$

where *At* was the absorbance value of the treated cells, *Ab* was the absorbance of CCK-8 only, and *Ac* was the negative control which included cells and CCK-8 only. Two types of controls were used: the media control consisted of cultured cells in 10% (*v*/*v*) FBS containing medium alone and the vehicle control consisted of cells in 10% (*v*/*v*) FBS containing medium, to which 10 μL of RPMI-1640 media without FBS was added. However, as both controls did not cause cytotoxicity, the media control was used to calculate cell viability.

Cell morphology was analysed at 48 h using a Nikon Eclipse TS100 (Nikon, Tokyo, Japan) phase contrast inverted microscope and the images were captured using a Nikon DS-Fi1digital camera.

#### 2.2.10. Statistical Analyses

Extractions were performed in triplicate and means ± standard deviation (SD) were assessed with the one-way Analysis of Variance (ANOVA) and Tukey’s *Post Hoc* Multiple Comparisons test using the IBM SPSS Statistics 24 program (IBM Corp., Armonk, NY, USA). Differences in means were considered statistically significant at *p* < 0.05. Correlations and their significance were determined using the Microsoft Excel 2016 (Microsoft Corp., Seattle, WA, USA) and Principle Component Analysis (Minitab 17.1.0, Sydney, NSW, Australia).

## 3. Results

### 3.1. Effect of Solvent on the Extractable Yield and the Dry Mass Yield

Extractable yield and dry mass yield were calculated for each extract. The extractable yields ranged from 4.4 g/100 g for the ethanol extract to 15.5 g/100 g for the aqueous extract while the dry mass yield ranged from 3.7 g/100 g for the ethanol extract to 13.1 g/100 g for the aqueous extract (Table 2). For both the extractable yield and the dry mass yield, the values were substantially higher for the aqueous extracts than for the organic solvents, whether they were mixed with water or not. Furthermore, the extractable yield (before drying) was higher than the dry mass yield (after freeze drying) for all extracts. The observed loss during the drying process was highest for the 70% ethanol extract (−31%) followed by the methanol extract (−28%) and the butanol extract (−20%). The aqueous and ethanol extracts had the same loss (−15%), while the lowest lost was for the 50% methanol extract (−11%).

### 3.2. Effect of Solvents on the Content of Bioactive Compounds

#### 3.2.1. Trypsin Inhibitors

**Figure 2:**
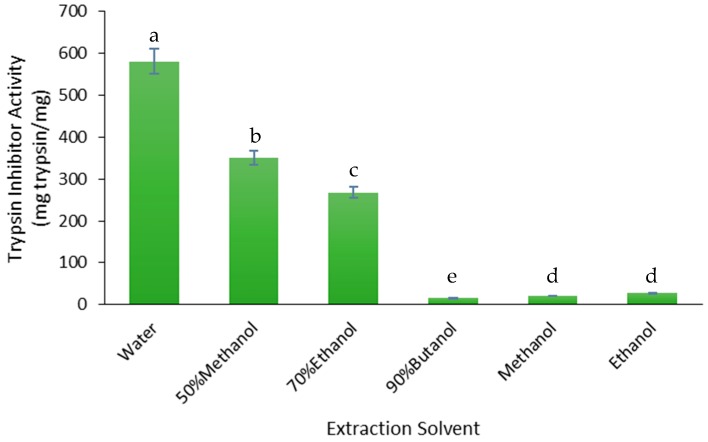
Effect of solvent on the trypsin inhibitor activity (TIA) of the FD crude extracts. The values are the means ± SD of three replicate extractions for each solvent. Columns not sharing the same letter are significantly different at *p* < 0.05.

The yield of trypsin inhibitors was measured as the trypsin inhibitor activity (TIA) of the FD crude extracts (Figure 2). The water extract had the highest TIA (581.4 mg/mg) and the yield decreased relative to the concentration of water in the water and low alcohol mixtures: −40% for the 50% methanol (≈50% water) extract, −54% for the 70% ethanol (≈30% water) extract and −97% for the water-saturated butanol (≈10% water) extract. The 100% methanol and 100% ethanol extracts had much lower TIA values than the water extract, −96% and −95%, respectively (Figure 2). Therefore, water was the best solvent for extracting trypsin inhibitors from the defatted Gac seed kernel powder.

#### 3.2.2. Saponins

**Figure 3:**
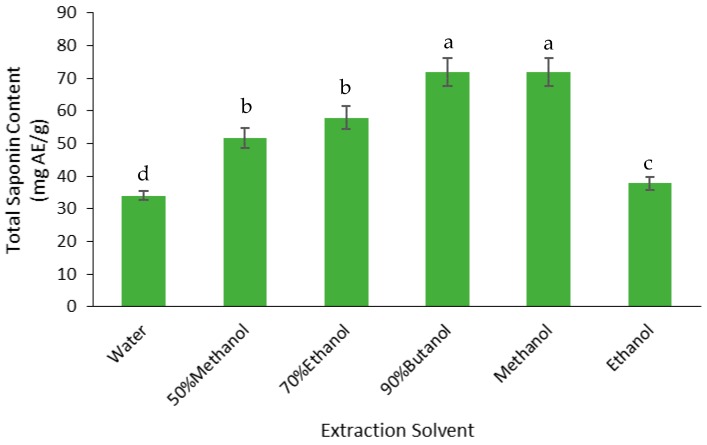
Effect of solvent on the total saponin content (TSC) extraction from Gac seed. The values were the means ± SD of three replicate extractions for each solvent. Columns not sharing the same letter were significantly different at *p* < 0.05.

The yield of saponins was measured as the total saponin content (TSC) of the FD crude extracts (Figure 3). The highest TSC was found in the butanol and methanol extracts, which were 24% higher than the TSC in the 50% methanol and 70% ethanol extracts, 48% higher than the ethanol extract, and 53% higher than the water extract. Therefore, butanol and methanol were the best solvents for extracting the saponins from the defatted Gac seed kernel powder.

#### 3.2.3. Phenolics

**Figure 4:**
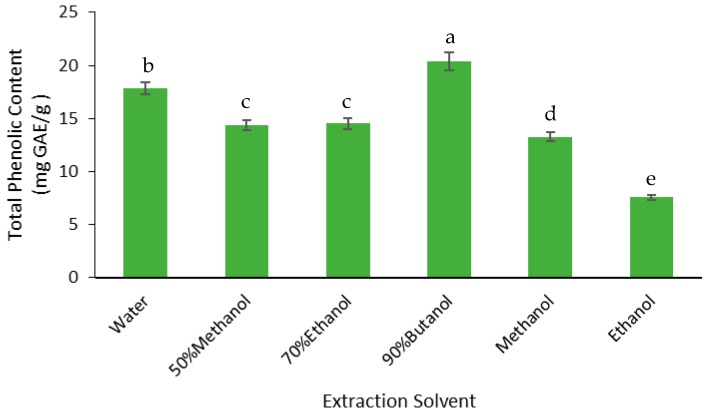
Effect of solvent on the total phenolic content (TPC) of the FD Gac seed crude extracts. The values were the means ± SD of three replicate extractions for each solvent. Columns not sharing the same letter were significantly different at *p* < 0.05. GAE, Gallic acid equivalents.

The yield of phenolics was measured as the total phenolic content (TPC) of the FD crude extracts (Figure 4). The highest TPC was found in the butanol extract; the TPC in this extract was 13% higher than the water extract, 29% higher than the 50% methanol and 70% ethanol extracts, 35% higher than the methanol extract, and 63% higher than the ethanol extract. Therefore, butanol extracted the highest phenolics from the defatted Gac seed kernel powder.

### 3.3. Effect of Solvents on Antioxidant Activity

**Figure 5:**
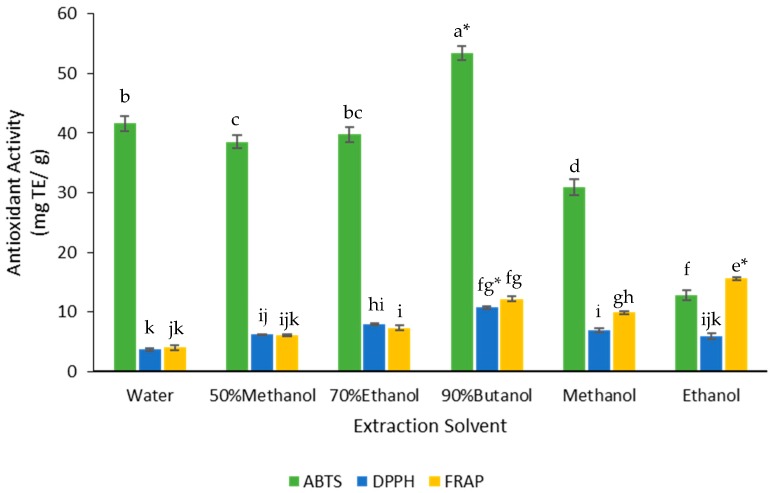
Effect of solvent on the antioxidant capacity of the FD crude extracts. The values were the means ± SD of three replicate extractions for each solvent. Columns not sharing the same superscript letter were significantly different at *p* < 0.05. * indicated the highest antioxidant activity. TE, Trolox equivalents.

The antioxidant activity of the extracts was measured using three assays: DPPH, ABTS, and FRAP. The butanol extract gave the highest values for ABTS and DPPH assays and the ethanol extract gave the highest value for FRAP assay (Figure 5). These two extracts produced significantly higher antioxidant activity based on the ABTS, DPPH, and FRAP assays (Figure 5).

### 3.4. Effect of Extraction Solvent on Cancer Cell Viability

**Figure 6:**
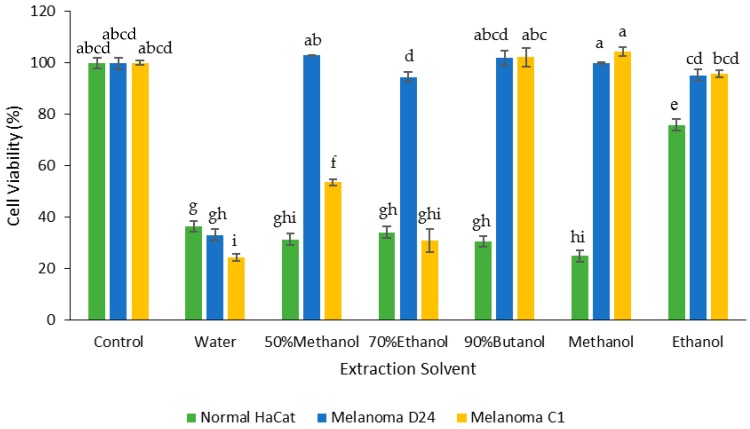
Effect of the FD crude extracts prepared with the different solvents on the cell viability of normal (HaCat) and melanoma (D24 and C1) cell lines after 48 h treatment. The values were the means ± SD of three replicate extractions for each solvent. Columns not sharing the same letter were significantly different at *p* < 0.05.

The FD crude extracts were tested for cell toxicity using two melanoma cell lines (D24 and C1) and a normal keratinocyte line (HaCat). The water extract was the most cytotoxic towards both cancer cell lines compared to the other extracts (Figure 6); the extract decreased the cell viability by 67% for D24 and 75% for C1 melanoma cells. The 70% ethanol and 50% methanol extracts also showed cytotoxic activity towards the C1 melanoma cells, decreasing their viability by 69% and 46%, respectively, while the butanol, methanol, and ethanol extracts had no effect. Besides the water extract, no other FD crude extract had any effect on the viability of the D24 melanoma cells.

In contrast, all the FD crude extracts decreased the cell viability for the normal keratinocytes (HaCat). Except for the ethanol extract, which only decreased the HaCat cell viability by 24%, all the extracts reduced the viability by between 64% (water) and 75% (methanol).

**Figure 7:**
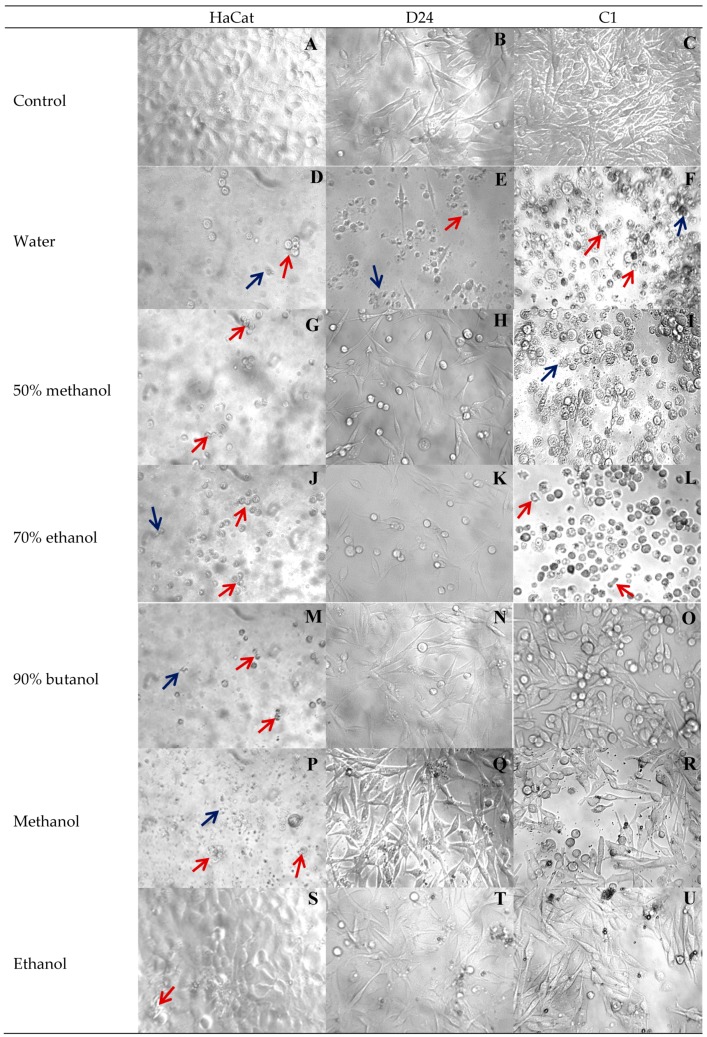
Morphological effects of different extraction solvents of Gac seed on HaCat (control) and melanoma D24 and C1 cell lines observed under a phase contrast microscope after 48 h treatment. Cytotoxicity is indicated by red arrows pointed to condensation and detached cells; blue arrows pointed to apoptotic bodies. Magnification: 100×.

These results were consistent with the cellular changes observed using a phase contrast microscope. As seen in Figure 7, the control untreated HaCat keratinocytes were firmly attached with flattened oblong shapes (Figure 7A), the D24 melanoma cells were lightly attached with advanced elongations (Figure 7B), and the C1 melanoma cells were firmly attached and tightly packed with elongated processes (Figure 7C). In comparison to the untreated cells (Figure 7A–C), exposure of the cells to the various Gac seed extracts induced typical changes of cell death, such as cytoplasmic condensation, detached cells (red arrows in Figure 7), and cell disruption, to form apoptotic bodies (blue arrows in Figure 7).

The cellular changes observed with the water extract (Figure 7D–F) were consistent with the viability results (Figure 6) in that signs of morphological changes were seen for all three cell types. The 50% methanol and 70% ethanol extracts also caused substantial morphological changes to the C1 melanoma cells (Figure 7I,L) and HaCat cells (Figure 7G,J). The butanol and methanol extracts also caused substantial morphological changes to the HaCat cells (Figure 7M,P). In contrast, the ethanol extract had the least effect on the morphology of all three cells (Figure 7S–U) akin to controls.

The most sensitive cells were HaCat keratinocytes, while the least sensitive were the D24 melanoma cells, which had the least morphological changes when treated with all the solvent extracts, except for the water extract (Figure 7).

### 3.5. Correlations between Extract Yields, Bioactive Compounds, Antioxidant Activity, and Cancer Cell Viability across the FD Crude Extracts

**Figure 8:**
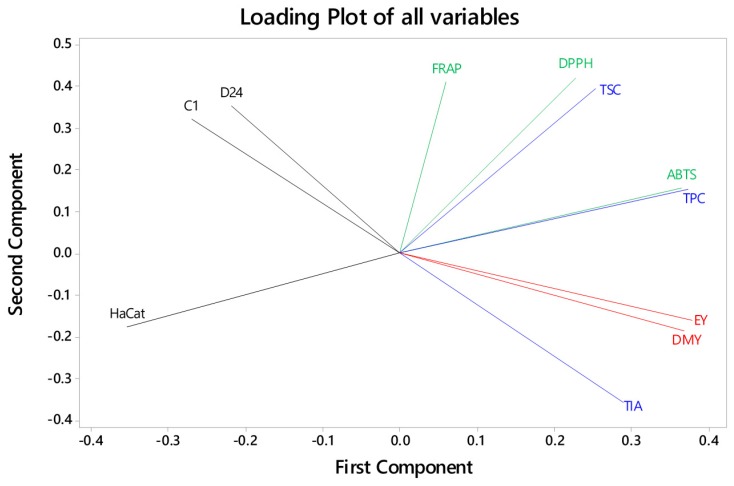
Correlations between extraction yields (red lines), bioactive compounds (blue lines), antioxidant activity (green lines), and cell viability (black lines) from Gac seed crude extracts.

TIA of the extracts was strongly and positively correlated with the extractable yield and the dry mass yield (Table 3, Figure 8). Essentially, the more material extracted, the higher the TIA, with the water extract having the highest values for both. TIA and extracted yields were also negatively correlated with the FRAP antioxidant activity and the viability of the C1 melanoma cells (Table 3, Figure 8). For example, almost 85% (*r*^2^) of the variability in the C1 cell viability could be explained by variability in the TIA. In contrast, the C1 cell viability was positively correlated with the FRAP antioxidant activity. Therefore, the negative effect of the water extract on the C1 cell viability may be related to its high TIA but not FRAP activity.

There were positive correlations between the TSC with DPPH antioxidant activity, and TPC with ABTS antioxidant activity (Table 3, Figure 8). However, there were no significant correlations between these bioactive compounds and any other variables, including the effects of the extracts on cytotoxicity for both C1 and D24 cancer cells (Table 3, Figure 8). Furthermore, there were no other significant correlations between any of the other measured parameters.

## 4. Discussion

Gac seeds have long been used in traditional medicine as a remedy for numerous conditions. Several bioactive constituents have been identified in Gac seeds, such as trypsin inhibitors, saponins, and phenolics. In this study, the extraction of these bioactive constituents with different solvents was investigated and their relationships with antioxidant activity and anticancer potential were explored. The results revealed that the water extract had the highest anticancer potential, which may be related to its high content of trypsin inhibitors but not to its antioxidant activity.

The water extract from defatted Gac seeds had the highest anticancer potential, reducing the growth of the melanoma cells compared to the control. This is consistent with the finding that a water extract from Gac seeds was the best extract for supressing the migration and invasion of a breast cancer cell line [18]. Water extracts from Gac aril have also been shown to suppress the viability of colon, liver [17], and melanoma [41] cancer cell lines. Compared to the previous study [17], where the Gac aril water extract at 1.24 mg/mL inhibited colon and liver cancer cells by 38 and 45%, respectively, in the present study, the water extract at the much lower concentration of 0.2 mg/mL had a significantly higher anticancer activity of 67% and 75% for the D24 and C1 melanoma cells, respectively. This suggests that water is not only safe and inexpensive, but it is also a highly efficient solvent for extracting compounds with anticancer potential from Gac, especially Gac seeds. The use of water as the extraction solvent also means that the methodology is widely accessible to more people, particularly in rural and underdeveloped countries that do not have access to organic solvents and processing facilities.

The water extract also had the highest TIA value, which was likely due to the presence of trypsin inhibitor proteins because Gac seeds are known to contain trypsin inhibitor peptides that are soluble in buffered aqueous solvents [31,33,42]. The strong inverse correlation between TIA and the viability of the C1 melanoma cells also suggests that trypsin inhibitors were involved in the water extract’s anticancer potential against these cells. The known Gac seed trypsin inhibitors have a low molecular weight of 3–4 kDa with a compact cyclic conformation [6,21], which can make it easy for them to penetrate into cancer cells and illicit cytotoxicity [43]. However, this is also consistent with the known anticancer potential of bigger proteins from Gac aril (35 kDa) [17] and other seeds, such as soybeans [44], which has been studied extensively [26,45,46,47].

However, although the water extract was the only extract to decrease the viability of the D24 melanoma cells, an inverse correlation between TIA and the viability of the D24 cells was not seen (*p* = 0.07) mainly because the 50% methanol and the 70% ethanol extracts had no activity against this cell line while they had activity against the C1 melanoma cell line. This could be due to the D24 cells, which have a mutated BRAF oncogene, being more resistant [48] to the presence of trypsin inhibitors, and therefore, they could only be killed when the trypsin inhibitors reach a high enough concentration, for example, in the water extract compared to the lower concentrations in the other extracts. However, it could also be that the D24 cells, and maybe also the C1 cells, were affected by other water soluble constituents of the Gac seeds, for example cyclotides which do not have trypsin inhibition activity [6]. For example, although many Gac seed trypsin inhibitors are cyclotides, MCoCC-1, a 3.3 kDa cyclotide unique to Gac seeds, which does not have trypsin inhibitor activity, has been shown to exhibit high cytotoxicity against the human melanoma MM96L cell line [6]; cell survival was decreased 43% in the presence of 2 µM MCoCC-1. The TIA is a measure of trypsin inhibitors, which may be cyclotides because they are found in Gac seeds [21], but other cyclotides are also present in Gac seeds that are purported to have anticancer potential [6,22]; however, these may not have antitrypsin activity and therefore would not be measured by the TIA.

The higher antioxidant activity of the butanol extract was likely due to its high content of saponin and phenolic compounds; this solvent had the highest TSC and TPC values among the extracts. There were strong positive correlations between saponins with DPPH antioxidant activity and phenolics with ABTS antioxidant activity, but neither saponins nor phenolic compounds were related to the FRAP antioxidant activity. Therefore, the extracted Gac seed saponins and phenolic compounds acted as antioxidants through the mechanism of scavenging the free radicals produced by DPPH and ABTS [39] rather than the reduction of oxidised intermediates in the FRAP chain reaction or through chelation [39]. A similar correlation was also reported by Chan et al. [49] for saponins and phenolic compounds extracted from defatted kenaf seeds. In that study, butanol was the most effective solvent for the extraction of saponins and phenolics. This solvent was non-polar enough to dissolve the saponin aglycones [50] and the phenolic rings [11], yet polar enough to also interact with the carbohydrate end of the saponin molecules and the carboxyl groups of gallic acid and p-hydroxybenzoic acid, which are reported to be present in Gac seeds [11]. Because butanol is classified by the FDA as a Class 3 solvent [51] with no known human hazards and approved for pharmaceutical applications, the use of it as an extraction solvent is regarded as less toxic and of low risk to human health.

Despite the high saponins, phenolics, and antioxidant activity of the butanol extract, it did not exhibit any anticancer potential against the two melanoma cell lines. This observation was not consistent with previous findings, which showed that one of the triterpenoid compounds found in Gac seed oil, karounidiol [20], had anticancer potential [19] and tumor inhibition effects [52]. This compound, likely a saponin, may not have been at a high enough concentration in the present extracts because the Gac seed kernels were defatted before they were extracted with the various solvents, which was consistent with a study by Le et al. [53], who found that Gac seed saponins were mainly associated with the fat component of the seeds.

## 5. Conclusions

Gac seeds, which are mostly considered as a waste product, could be a potentially useful source of anticancer candidates due to their cytotoxic activity against specific melanoma cells. The utilisation of Gac seeds to produce an anticancer product could reduce the waste burden on the environment and add value to the Gac fruit, which is cultivated in an increasing number of countries. This study found that water is suitable for the recovery of trypsin inhibitors and the preparation of an extract with significant anticancer potential. Butanol was found to be suitable to produce an extract enriched in saponins and phenolic compounds and with a high antioxidant activity. However, further studies are needed to fully understand what specific compounds are involved in the bioactivity of Gac seeds and their mechanism of action in order to better define their potential applications in the nutraceutical and pharmaceutical industries.

## Figures and Tables

**Table 1:** The trypsin inhibitor activity assay setup.

Component	Reagent Blank (a)	Standard (b)	Sample Blank (c)	Sample (d)
Deionised water (mL)	2	2	1	1
Trypsin solution (mL)	-	2	-	2
Diluted extract (mL)	-	-	1	1
BAPNA (mL)	5	5	5	5
Acetic acid (mL)	1	1	1	1
Trypsin solution after reaction inactivation (mL)	2	-	2	-

BAPNA (benzyl-DL-arginine-para-nitroanilide) was added to start the reaction and acetic acid for inactivation.

**Table 2:** Effect of solvent on the extractable yield (EY) and the dry mass yield (DM).

Solvent	EY (g/100 g)	DM (g/100 g)	Original Volume (mL)	Collected Volume (mL)	Yield Loss (%)
Water	15.5 ± 0.1 ^a^	13.1 ± 0.1 ^b^	400 ± 3	355 ± 0	15.3 ± 0.1 ^d^
50% Methanol	10.2 ± 0.0 ^c^	9.1 ± 0.3 ^d^	400 ± 3	348 ± 3	10.9 ± 0.2 ^f^
70% Ethanol	10.2 ± 0.0 ^c^	7.1 ± 0.4 ^e^	400 ± 3	340 ± 5	30.6 ± 0.3 ^a^
90% Butanol	6.9 ± 0.1 ^e^	5.5 ± 0.1 ^f^	400 ± 3	350 ± 0	19.9 ± 0.1 ^c^
Methanol	6.7 ± 0.2 ^e^	4.8 ± 0.2 ^g^	400 ± 3	330 ± 0	27.8 ± 0.2 ^b^
Ethanol	4.4 ± 0.0 ^g^	3.7 ± 0.0 ^h^	400 ± 3	345 ± 0	14.9 ± 0.0 ^e^

The values were the means ± SD of three replicate extractions for each solvent. All the values for EY and DM were compared to each other, the values for yield loss were compared separately and the values not sharing the same superscript letter for EY and DM, and separately for yield loss, were significantly different at *p* < 0.05.

**Table 3:** Coefficient of correlations (*r*) between yields, bioactive compounds, antioxidant activity, and cell viability across the Gac seed crude extracts.

	Yield		Bioactive Compound			Antioxidant Activity			Cell Viability		
	EY	DMY	TIA	TSC	TPC	ABTS	DPPH	FRAP	HaCat	D24	C1
EY	1.00										
DMY	0.98 ^†^	1.00									
TIA	0.96 ^‡^	0.97 ^‡^	1.00								
TSC	−0.42	−0.50	−0.61	1.00							
TPC	0.51	0.49	0.31	0.36	1.00						
ABTS	0.48	0.43	0.29	0.44	0.97 ^†^	1.00					
DPPH	−0.52	−0.58	−0.66	0.81 ^§^	0.37	0.46	1.00				
FRAP	−0.93 ^‡^	−0.89 ^§^	−0.88 ^§^	0.19	−0.48	−0.50	0.44	1.00			
HaCat	−0.42	−0.35	−0.22	−0.63	−0.73	−0.78	−0.28	0.63	1.00		
D24	−0.79	−0.80	−0.77	0.65	−0.28	−0.13	0.69	0.56	−0.01	1.00	
C1	−0.88 ^§^	−0.83 ^§^	−0.92 ^§^	0.53	−0.23	−0.26	0.50	0.83 ^§^	0.18	0.61	1.00

^†^*p* < 0.001, ^‡^ *p* < 0.01, ^§^ *p* < 0.05, EY: Extraction yield; DMY: Dry mass yield; TIA: Trypsin inhibitor activity; TSC: Total saponin content; TPC: Total phenolic content; ABTS: 2,2′-Azino-bis(3-ethylbenzothiazoline-6-sulfonic acid) assay; DPPH: 2,2-diphenyl-1-picrylhydrazyl assay; FRAP: ferric reducing antioxidant power assay.
